# Injectable contraceptive use in women with intellectual disability: narrative review and service evaluation

**DOI:** 10.1192/bjo.2025.786

**Published:** 2025-08-20

**Authors:** Marianne Bergman, Alaa Martin

**Affiliations:** Essex Partnership University Trust, Wickford, UK; Intellectual Disability, Hertfordshire Partnership Foundation Trust, Hatfield, UK

**Keywords:** Contraception, hormones, intellectual disability, physical health

## Abstract

**Background:**

Intellectual disability is defined as an IQ of 70 or below. Women with intellectual disability frequently experience menstrual distress leading to the use of hormonal medications such as depot medroxyprogesterone acetate (DMPA). Despite risks such as reduced bone mineral density (BMD) and weight gain, DMPA is widely used in this cohort, prompting investigation into its suitability and risks.

**Aims:**

A narrative review and local service evaluation were conducted to determine whether clinical management reflected recommendations in the literature.

**Method:**

PsycINFO and Medline were searched for articles post-1995 on contraception in menstruating women with intellectual disability. Contraceptive use in 100 randomly selected women was evaluated. Data were collected on physical health issues, general practitioner records were reviewed for contraceptive administration and risk discussions, and surveys assessed risk understanding and satisfaction.

**Results:**

The review identified 27 papers with higher DMPA use in the intellectual disability population compared to the general population, and specific BMD risks. The case series found 23 women with intellectual disability using DMPA, and revealed knowledge gaps in risk and monitoring, inappropriate use given individual risk, and poor proactive risk management.

**Conclusions:**

Findings indicate disproportionate DMPA use in women with intellectual disability, with inadequate clinical justification and risk awareness. Many women and carers were unaware of BMD risks, and DMPA alternatives were rarely considered. Individualised contraceptive management and closer review of DMPA use in this cohort is needed. Monitoring could include dual X-ray absorptiometry (DEXA) scans, vitamin D and calcium supplementation, and weight management. Further research is needed into higher DMPA use and risks within this population.

The World Health Organization (WHO) defines intellectual disability as an IQ of 70 or below.^1^ Women with intellectual disability experience inequality in both the rate of diagnosis and management of their physical health.^2^ They present as a varied population cohort, with physical health diagnoses and risks complicating psychiatric management. Management options include use of anti-epileptic drugs (AEDs) for both epilepsy and mood stabilisation, and anti-psychotic medication to treat underlying psychotic, mood and behavioural disorders – as well as management of any contributing physical health issues.^2^

Within the female cohort, menstruation can result in distress for both women with intellectual disability and their carers.^3,4^ Contributors to this distress include aversion to blood, dysmenorrhoea and menorrhagia, and premenstrual tension.^5,6^ Furthermore, some women can suffer from catamenial epilepsy or develop anaemia.^7^ Subsequently, many women with intellectual disability are prescribed hormonal methods to help manage their menstrual cycle.^8^ One of the most utilised methods is depot medroxyprogesterone acetate (DMPA).^9^ While there are non-hormonal management options for menstrual distress, such as educational initiatives,^10^ several papers published on contraceptive use within this cohort acknowledge that DMPA use is increased in the intellectual disability population – but there is no consensus on why.^3,8,11,12^ There are known risks associated with DMPA use that are not seen with other contraceptives such as the progesterone-only pill or implant. DMPA is associated with risks of reduced bone mineral density (BMD) and weight gain.^13^ Unfortunately both reduced BMD and weight gain are seen more commonly within the intellectual disability population. BMD can be lowered by reduced mobility, long-term use of proton pump inhibitors, raised prolactin (related to the use of antipsychotics), and AEDs, making this population more at risk.^14–16^ Awareness surrounding reduced BMD in this cohort, particular AED users, has increased in recent years, and recent papers have highlighted a lack of awareness of such risks within the user and carer population.^16,17^ Indeed, a recent audit was carried out on this within the patient cohort studied in this paper.^18^ Weight gain can be contributed to by poor diet related to food aversion and some genetic conditions such as Down syndrome or Prader–Willi syndrome.^10^ Studies agree that there is a lack of research on DMPA use in women with intellectual disability, who have an additional risk profile for its side effects.^14,19^

This study includes a narrative review paired with a local case series to explore the range of current literature and guidelines, as well as the scope of contemporary practice. Data will be analysed on a selection of the local intellectual disability cohort to determine a rough percentage of DMPA users and assess whether the benefits and risks of this medication are being accurately assessed and discussed. It aims to look at the numbers of users within a selection of patients, their reasons for use and whether successful. It also aims to look at whether the appropriate monitoring has been carried out for users, and whether there are any women who are found to have contraindications despite prescription. The overall focus is to see whether the literature reflects findings within this cohort. It seems prudent to investigate this area for a multitude of reasons. Important preventative measures may be missed by not considering the physical health impact of DMPA, such as vitamin D and calcium supplementation. DMPA may also be unwittingly contributing to weight gain and fracture risk in a cohort already at risk.

## Method

### Narrative review

A systematic search was undertaken using appropriate search terms from the PsycINFO and Medline databases (please see Table 1 for search terms). Inclusion criteria incorporated articles published since 1995, women with a diagnosed intellectual disability (IQ < 70) who were post-menarche but pre-menopause. Age cut offs were defined as 13–45 years, as menarche most commonly starts around 13, and early menopause is defined as the cessation of menstruation before the age of 45.^20,21^ International studies were included, including non-English language studies. 214 possible relevant articles were found using the keyword search. Titles of articles were assessed and 160 were excluded as irrelevant. Following this, 54 article abstracts were assessed, and of these, 27 articles had a full text review. Quantitative synthesis was undertaken to explore the rationale for each contraceptive choice, and to investigate any effort to discuss metabolic risks or any discussion of alternative approaches. Quantitative descriptive statistics were used to describe the distribution of different contraceptive methods with comparison to reference data from the general population.

### Service evaluation

A service evaluation was carried out investigating the use of injectable contraceptives in women with intellectual disability aged 13–45. Ethical approval was granted by the Integrated Governance Committee. 100 patients matching these demographics were selected at random from Hertfordshire Partnership Foundation Trust (HPFT) intellectual disability caseload lists, utilising a random number generator (www.random.org). A case note and shared care record review was undertaken to determine whether those selected were on a hormonal contraceptive method. The review was discussed with the patient, care team or next of kin over the phone or in clinic as appropriate – consent to participate in the study was asked of the patient at the time, or, if lacking capacity, of their next of kin, and recorded in an anonymised spreadsheet. Discussions took place either with both M.B. and A.M. present or M.B. and another resident doctor colleague present. Furthermore, consent was implied through completion of the survey discussed below, as specified in an accompanying letter. Caseload lists and case notes were taken from PaRIS, the clinical record system utilised across HPFT. Shared care records were reviewed utilising the PaRIS shared records platform.

A simple survey was designed by M.B. and A.M. and was sent by mail to selected women prescribed DMPA. An instruction letter was included with information on the purpose of the study and reason for correspondence, and advising that the survey be completed by the patient, family or carers depending on suitability. The survey asked for basic demographic information, then information on contraceptive use: the type of injectable contraceptive, reason for commencement (menorrhagia/contraception/other), length of use, any side effects and whether the completer was aware of the side effects unique to the depot. They were also asked whether it had worked for the reason prescribed, and the impact on bleeding pattern. Furthermore, they were asked whether there was a diagnosis of epilepsy, about mobility, anti-psychotic and AED use, and use of calcium and vitamin D supplementation. The survey also asked whether they had been offered 2-yearly reviews by their general practitioner (GP) as per UK Medical Eligibility Criteria (UKMEC) guidelines.^13^ They were advised to leave a contact number if they wished to discuss the survey or side effects in greater detail. Those who did not respond to the survey were telephoned and consent and survey answers taken over the phone.

Following the survey, the West Suffolk Shared Care record was accessed through PaRIS to find information on initiation of DMPA, administration frequency, discussion of risks and use of prior methods. Medications were reviewed to confirm whether participants were taking AEDs, vitamin supplementation or prolactin-raising antipsychotic medications. If this information could not be found, GP surgeries were emailed for provision of this information. Both shared care and PaRIS records were analysed to determine their age, body mass index (BMI) and, if available, calcium and vitamin D status, prolactin levels and lipid status.

Responders who had not been aware of additional risks of DMPA were contacted for further discussion and advised to make an appointment with their GP to review the DMPA method suitability, in comparison to alternative options. An email was sent to the GP to request a review if indicated. Women with intellectual disability who were found to be prescribed DMPA where UK Medical Eligibility Criteria (UKMEC) guidelines recommended that risks outweighed the benefits (score of >2) were contacted and an email sent to their GP recommending a review.^13^

## Results

### Narrative review

**Fig. 1:**
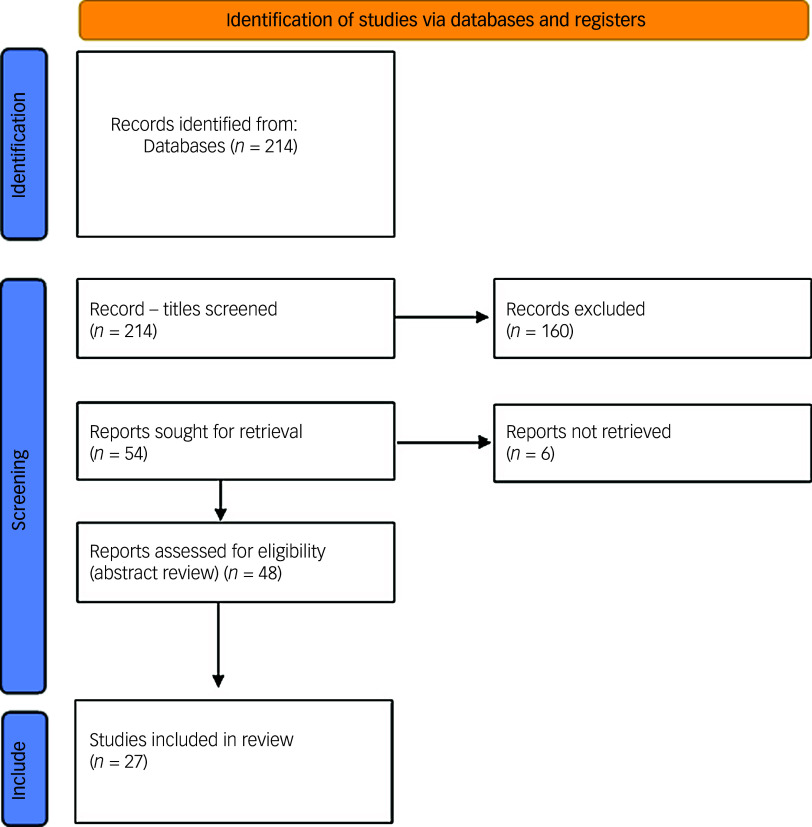
PRISMA 2020 flow diagram showing the process of the identification of suitable papers for assessment.^22^

Of the 27 papers reviewed, the majority were observational studies, with one population-based cohort study and two systematic reviews (Fig. 1).

In terms of the contraceptive method prescribed, while there was slight variation in numbers of users between papers, a frequent finding was that DMPA use was higher in the intellectual disability population than the general population (Table 2).^11,14,23–27^ Furthermore, while anecdotally there have been some uncertainties around the use of long-acting intrauterine methods, Mirena was found to have therapeutic benefit.^3^

**Fig. 2:**
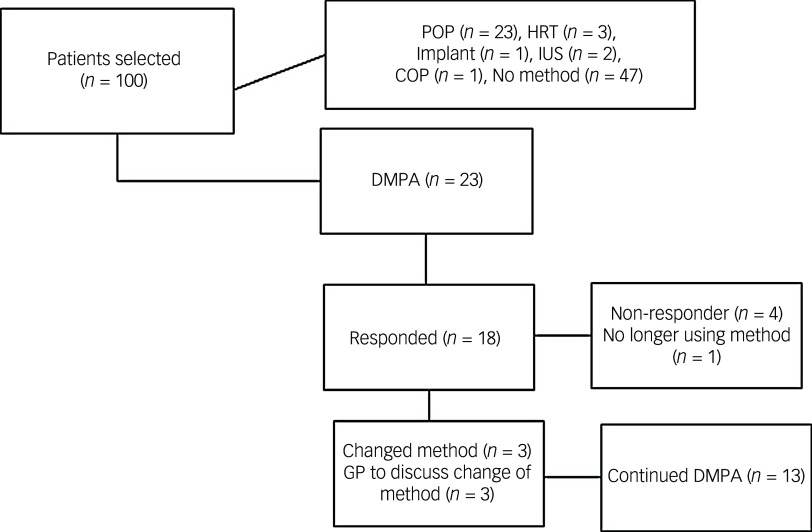
Flow diagram to indicate the outcome of 100 women with intellectual disability selected. POP, progesterone only pill; HRT, hormone replacement therapy; IUS, intrauterine system; COP, combined oral pill; DMPA, depot medroxyprogesterone acetate; GP, general practitioner.

The reasons for the use of contraceptives in the intellectual disability population were evaluated. The most common reason was for suppression of menstruation.^23,27,28^ Additional reasons included menstrual-related symptoms in autism, pre-menstrual syndrome and behavioural changes related to the menstrual cycle.^5,19,20^ In terms of care-related reasons, contraceptives were cited as helping with menstruation management as they were convenient, effective and helped with hygiene.^12,24,29^ Pregnancy prevention was rarely the main reason for use.^4^ Regarding menorrhagia management, Albanese and Hopper made specific recommendations that extended combined oral contraceptive pill would be the simplest first choice for menorrhagia, followed by Norethisterone 5 mg TDS – not finding DMPA to be a suitable first-line option.^11^ Brown et al recommended NSAIDs (non-steroidal anti-inflammatory drugs) and tranexamic acid as first-line, followed by non-invasive contraceptive methods.^30^

Women with intellectual disability were generally not included in discussions regarding their contraceptive choices.^4,8,15,20^ No clear reasons were given for this – but themes included lack of engagement with the GP and lack of contraceptive education to aid capacity. This seems to fit with a paternalistic model. Brown et al noted this and felt that women with intellectual disability needed to be included in informed consent discussions wherever possible.^30^

Several papers discussed whether there was a pharmacological reason women with intellectual disability should be given DMPA at higher rates than the general population – but overall no clear reason was identified.^8,14,20^ Price does not seem to be a credible factor. Current British National Formulary (BNF) pricing suggests that the cost of one DMPA injection is around £6 (not including the cost of a nurse appointment to administer it). DMPA methods which can be delivered by a women with intellectual disability, her caregiver or a family member are £6.90 a dose – but a 3-months supply of the common progesterone-only pill desogesterel is £2.78, and a first-line 3-month supply of a generic combined pill is £1.81.^31^ Furthermore, compared with other longacting reversible contraceptives (LARCs), such as the implant or intrauterine system or device (IUS/IUD), DMPA is not as cost-effective.^31^ Hence this does not offer an explanation.

**Table 1:** Search terms from the PsycINFO and Medline databases used for the narrative review

	Search terms	Search results
S14	S5 AND S13	(126)
S13	S6 OR S7 OR S8 OR S9 OR S10 OR S11 OR S12	(144 690)
S12	Definition ‘Cerebral Palsy’	(7290)
S11	Definition ‘Down’s Syndrome’	(7095)
S10	Definition ‘Fragile X Syndrome’ O Definition ‘Prader Willi Syndrome’ OR Definition ‘Williams Syndrome’	(4038)
S9	Definition ‘Autism Spectrum Disorders’ OR Definition ‘Neurodevelopmental Disorders’ OR Definition ‘Rett Syndrome’	(61 808)
S8	Abstract (‘intellectual* disabilit*’ or autism or autistic or ‘down* syndrome*’ or neurodevelopmental or ‘Fragile X’ or ‘Rett* syndrome’ or ‘William* syndrome’ or ‘Prader-Willi*’ or phenylyketonuria or ‘cerebral palsy’ or ‘DiGeorge syndrome*’ or ‘22q11 deletion*’)	(106 743)
S7	Abstract (‘learning disorder*’ or ‘learning disabilit*’)	(18 107)
S6	Definition ‘Learning Disorders’ OR Definition ‘Learning Disabilities’	(28 841)
S5	S1 OR S2 OR S3 OR S4	(21 722)
S4	Abstract (‘birth control’ or condom*)	(12 932)
S3	Definition ‘Condoms’ OR Definition ‘Diaphragms (Birth Control)’ OR Definition ‘Intrauterine Devices’ OR Definition ‘Oral Contraceptives’	(7566)
S2	Definition ‘Birth Control’ OR Definition ‘Contraceptive Devices’	(5043)
S1	Abstract contracepti*	(9023)

**Table 2:** Contraceptive method by % of total female population of childbearing age^4,8,9,21^

Population and source	DMPA	Sterilised	Oral contraception	IUD/IUS	No use
General population					
Sexual and Reproductive Health Services, England 2022–2023	7	1	27	26	–
LD populations					
Servais et al (2002).^9^	**17.6**	22.2	18.4	1	40.8
Van Schrojenstein (2011).^4^	**28**	–	63	–	20
McCarthy (2011).^8^	**34.2**	–	40	–	–

DMPA, depot medroxyprogesterone acetate. IUD/IUS, intrauterine device/intrauterine system.

**Table 3:** Table indicating cardiovascular risks in 5 women with intellectual disability using DMPA with risk factors

Patient number	BMI>30	Low HDL	High total cholesterol	Smoker	Use of SGA	Diabetic
1	X	X			X	
2		X	X		X	
3	X	X		X	X	
4	X			X		X
5	X	X				X

DMPA, depot medroxyprogesterone acetate; BMI, body mass index; HDL, high-density lipoprotein; SGA, second generation antipsychotic. X represents the presence of a risk factor.

Risks of DMPA were considered. In Quint et al’s systematic review, it is generally accepted that the intellectual disability population have a higher risk of BMD than the average population.^15^ There were also concerns regarding the impact of weight gain, which could complicate transfers for those with mobility issues and impede independence by reducing mobility.^26,30^ A variety of investigations to quantify these risks were considered by various authors, including DEXA scanning, supplemental vitamin D and calcium for all users, weight-bearing exercises and ‘add back oestrogen’ for those utilising DMPA.^11,27,30,32,33^ Furthermore, the UKMEC requires 2-yearly risk–benefit reviews for those on DMPA to ensure BMD and weight risks are regularly considered for users.^13^ It was acknowledged that there is a lack of specific research into DMPA, reduced BMD and weight gain in this population – despite a higher rate of use and increased risk in this population at baseline.^14,15,23^

### Service evaluation

One-hundred female women with intellectual disability aged 13–45 and living in the community were selected at random and their records analysed. The mean, median and modal ages were 37, 30 and 18, respectively. Thirty-two of the women had mild intellectual disability (IQ 50–70 ), 27 moderate (IQ 35–49) and 38 severe (IQ < 35), with three uncategorised. All women resided in North West and Mid Essex, in the UK. Data on ethnicity was not considered due to lack of consistent documentation.

Of the 100 women with intellectual disability analysed, 23 were found to be utilising DMPA. Of the remaining women with intellectual disability, 23 were found to be using desogestrel 75mcg, 3 utilising HRT (Hormone Replacement Therapy), 1 fitted with the progesterone-only implant, 2 fitted with Mirena coils and 1 prescribed the combined oral contraceptive pill. The mean, median and modal ages were 31, 30 and 40, respectively. The ages of DMPA users were not significant compared with the cohort utilising other methods (*p* = 0.570). There were also no significant difference in rates of mild, moderate and severe intellectual disability (*χ* ^2^ = 0.293).

Of the DMPA cohort, one woman was excluded as they were no longer prescribed this. Seven women had tried a different method prior to DMPA which they had not tolerated or which had not been suitable. Eleven women with intellectual disability were on an AED (which reduces BMD) and, of these, four were on enzyme inducing AEDs. One woman utilised a PEG feeding tube, and carers found administering crushed tablets difficult. One woman found it difficult to swallow tablets so DMPA was felt to be easier. Hence, of the 22 assessed, 13 had a reason for its prescription over other methods (Table 3).

### Cardiovascular and weight risks

Eleven of the cohort had a BMI >25. Of these, seven were obese. Five women with intellectual disability were found likely to meet UKMEC category 3 for DMPA – due to ‘multiple risk factors for CVD (such as smoking, diabetes, hypertension, obesity and dyslipidaemias)’. In the UKMEC guidelines, a low HDL (high-density lipoprotein) was specifically linked to increased cardiovascular risk in users of DMPA.^13^

### Bone mineral density risks

Three women with intellectual disability were found to have low calcium and four to have low vitamin D on recent blood tests. Only one with low calcium was on a regular calcium supplement, and half of those with low vitamin D were on replacement. Three women were unable to tolerate blood tests so had no recent results. Of the 22, 1 was PEG (percutaneous endoscopic gastrostomy), fed in line with regular reviews from the dietician to ensure their macro- and micronutrient requirements were met; 10 were on no calcium or vitamin D supplementation, 5 were on dual calcium and vitamin D and 6 were on vitamin D supplementation alone. In terms of weight-bearing exercise and limitations to this, nine women with intellectual disability utilised a wheelchair and one utilised a roller to mobilise (Fig. 2).

### Monitoring, risk awareness and efficacy

Of the 22 women surveyed, full responses were received from 18. Three women answered survey questions themselves, with the rest of the surveys answered by care staff or family members. Of these, 13 reported mandatory 2-yearly checks from the GP. Only nine of these women with intellectual disability and/or carers were aware of the risks of reduced BMD and increased weight. Ten of the 22 women or carers were happy with the overall risk–benefit after discussion.

Of the women surveyed, six had an email sent to the GP to discuss consideration of a new method. This included possible UKMEC 3 scores, concern about the risks and a general desire to discuss other methods. Three were changed to the progesterone-only pill, with the three other conversations outstanding.

## Discussion

The purpose of this study was to carry out a service evaluation of the female menstruating intellectual disability cohort within HPFT services in Essex and determine whether contraceptive prescription practice was appropriate, safe and reflected the findings of the narrative review. As previously discussed, it is important to examine contraception in this population for a number of reasons. First, 20–25% of people with an intellectual disability have epilepsy and take AEDs, which can interact with contraceptives.^34^ Second, those with intellectual disability are more likely to have concurrent health problems, and health problems picked up later than those in the general population, particularly those unable to communicate verbally.^35^ NICE also recommends reducing polypharmacy wherever possible.^35^ Hence, considering the impact of any long-term medication in this group is vital.

This paper aimed to examine the literature and then assess a patient group to see if findings in the literature matched granular patient-level data. The percentage of 22% of DMPA users within our population was comparable to that within the wider literature, as were the reasons for utilising this method, with 91% using for menstruation management.^4,9^ This work aimed to examine patient and carer knowledge of the increased risks of DMPA use and adherence to UKMEC guidelines, something that was not found in the current literature. Of 22 women with intellectual disability investigated and 18 surveyed, only 53% of the women or their care teams were aware of the risks of reduced BMD and increased weight, and nearly a third (27%) were subsequently referred to their GP for discussion of alternate methods. This appears to be in keeping with the lack of awareness about reduced BMD found in prior papers on AEDs.^16–18^ A subset of those who answered the survey was concerned about risks and, if educated on risks, asked about other medications. Furthermore, 59% of women had not tried another method, despite recommendations that DMPA is avoided in this cohort as a first-line treatment.^11,20^ During discussions with carers and family members, ease of use was often mentioned, as was a fear over AED use resulting in oral methods not working properly (despite misconceptions, many AEDs do not interact with oral tablets). Another issue seems to be compliance: carers and families often mentioned that medications could be difficult to administer, and that the injection worked well for cessation of menstrual bleeds so was the easier option. This is in keeping with findings of prior literature.^8,11^ It was striking that in the literature, many women were not included in discussions around their contraception use, and this was reflected in our review. Documented discussions often found that the decision had been made by the care staff or the primary care team. It seems that a paternalistic model has been upheld in this particular area of practice.

We acknowledge limitations with this study. The survey was relatively brief and focused on practical questions around DMPA use. All but one of the surveys were completed by the care team rather than the service user themselves. Moving forwards, the authors feel that the best way to collect this data would be to carry out the survey in a patient-facing consultation, with the use of easy-read-format and communication aids if appropriate. Many women in this cohort may have varying levels of capacity regarding their healthcare – however, capacity must be assessed on a decision-specific basis. Research suggests that discussions around contraceptive use in this population often lack transparency, with limited exploration of alternative options or full disclosure of potential risks.^4^ Ensuring that capacity and best-interests decisions are robust, evidence-based and include the perspectives of the individual wherever possible is essential to safeguarding autonomy and improving contraceptive and menstrual management within this cohort. It would also be useful to ensure the survey captures the role of the next of kin or staff member completing the survey, to examine whether there is a difference in knowledge or attitudes between different roles.

The authors acknowledge that in order to get a wider perspective on DMPA use within this cohort, the psychological impact of both menstrual distress and DMPA use needs to be considered. As with all hormonal contraceptives, side effects can include mood and anxiety symptoms, so it would be useful to consider this in more detail. Furthermore, this was a small, single-centre study covering one mental health trust, with a small cohort of patients. While multiple primary care clinical commissioning groups were captured, moving forwards it would be beneficial to include a wider geographical location for a more varied demographic and higher number of patients. This would ensure greater generalisability of findings. In addition, because of the retrospective nature of the work, there was variability in availability of primary care records on DMPA prescriptions. There was also some difficulty for survey completers in recalling specific dates or reasons for commencing the medication. This was particularly the case for those women who had been on DMPA for many years, which may mean we are missing key information on those who have had the longest use. While we looked at GP records, often the records only went back for a few years. These factors made it difficult to determine how long the women had been on this method, hence the lack of inclusion of this information in the results.

Further research looking at DMPA use in this specific cohort appears necessary. Women with intellectual disability are high users compared with the general population yet have increased risk factors for additional side effects compared with other methods. Hence there is a discrepancy here. The authors found a lack of clear data regarding BMD and weight risks, despite this being flagged as an area of concern in the literature since the 1990s. It seems vital that the reported ‘need’ for DMPA is examined. Future work could examine how often non-medical interventions are used to manage menstrual distress before hormonal options are looked at, and what the barriers are for offering this to more women. For those who continue to use DMPA there is a need for formal guidance on management, including vitamin D and calcium supplementation and monitoring, as well as consideration of DEXA scanning, body weight monitoring and whether there should be specific cautions or contraindications within this group – that is, AED use, poor mobility or BMI. In 2022, it was found that the number of deaths from ischaemic heart disease in the intellectually disabled appeared to be increasing.^33,34^ Indeed, high BMI is associated with increased mortality overall, and avoidable deaths within the intellectual disability cohort, and should not be overlooked.^7^ Further research could be carried out to look at the psychological impact of DMPA use. The impact of hormonal changes within the intellectual disability cohort within the menopause is increasingly recognised – hence utilising a medication which induces a menopausal state is likely to result in some of the same issues. This has not been covered in this paper but is a recognised area of interest.

In our own clinical practice we aim to include specific questions around DMPA use and risk assessment in physical health reviews. We aim to invite discussion around different contraceptive methods, even if women with intellectual disability have been on a method for many years. It is important not to assume that options such as the progesterone implant or IUS will be rejected as they offer the menstrual benefits of DMPA without the risk. Furthermore, we will aim to review vitamin D and calcium status more closely within the DMPA cohort, as well as monitor weight. We must consider the fact that a subset of women with intellectual disability would be unable to tolerate a DEXA scan or monitoring regular blood tests.

Ultimately, the higher use of DMPA in this cohort, found in both the narrative review and evaluation of our population, is of concern. DMPA use was not in keeping with Faculty of Sexual and Reproductive Health UKMEC recommendations – reviews and appropriate tests deemed important for monitoring were not consistently done.^13^ Furthermore, many carers and women with intellectual disability were unaware of the added risks of weight gain and reduced BMD.^5^

We aim to re-review this cohort in 6–8 months’ time to determine whether there has been a change in DMPA use compared with other methods. We aim to carry out a more detailed survey to assess concerns associated with both DMPA and other methods.

## Data Availability

The data that support the findings of this study are available on request from the corresponding author, M.B. The data are not publicly available to protect research participant privacy.
